# GUM: Gum Understanding Mission—A Serious Game to Improve Periodontitis Literacy Among University Students

**DOI:** 10.3390/dj14040242

**Published:** 2026-04-18

**Authors:** Franklin Parrales-Bravo, Hugo Arias-Flores, Luis Caguana-Alvarez, Miguel Dávila-Medina, Carolina Parrales-Bravo, Leonel Vasquez-Cevallos

**Affiliations:** 1 Artificial Intelligence Research Group, Universidad Bolivariana del Ecuador, Km 5 ½ vía Durán—Yaguachi, Durán 092405, Ecuador; franklin.parralesb@ug.edu.ec; 2 Grupo de Investigación en Inteligencia Artificial, Facultad de Ciencias Matemáticas y Físicas, Universidad de Guayaquil, Guayaquil 090514, Ecuador; luis.caguanaa@ug.edu.ec (L.C.-A.); miguel.davilam@ug.edu.ec (M.D.-M.); carolina.parralesb@ug.edu.ec (C.P.-B.); 3 Faculty of Computer Science, Complutense University of Madrid, Av. Séneca, 2, 28040 Madrid, Spain; 4 Centro de Investigación de Ciencias Humanas y de la Educación (CICHE), Universidad Tecnológica Indoamérica, Quito 170103, Ecuador; 5 Facultad de Ingenierías, Arquitectura y Ciencias de la Naturaleza, Universidad ECOTEC, Samborondón 092301, Ecuador

**Keywords:** educational game, oral health education, periodontitis literacy, adaptive learning, gamification, digital intervention

## Abstract

**Background/Objectives:** Periodontitis represents a significant global health burden, yet preventive health literacy remains critically low among emerging adults—a developmental stage where lifelong health behaviors crystallize. This study evaluated the effectiveness of the GUM (an acronym of Gum Understanding Mission) game, an interactive gamified digital tool incorporating AI-informed or manual feedback, for improving periodontitis literacy among tenth-semester Software Engineering students at the University of Guayaquil. **Methods:** In a controlled pre-test/post-test experiment, 50 participants were randomly assigned to either the GUM game intervention or a traditional lecture. Both groups completed identical knowledge assessments immediately before and after their respective 50-min instructional sessions. The GUM game featured adaptive questioning, immediate elaborated feedback, and comprehensive performance analytics, while the control group received instructor-led didactic instruction with a subsequent question-and-answer session. **Results:** The GUM group improved from a baseline of 21% to 94% correct responses, while the lecture group increased from 22% to 67% (p<0.001). Error reduction was 74% in the GUM group versus 45% in the control group. However, the study’s scope is currently limited to a single, digitally literate cohort, and knowledge retention over time was not assessed. **Conclusions:** These findings suggest that a self-directed, feedback-driven serious game can substantially outperform traditional methods in fostering periodontitis literacy within this population. Further research is needed across diverse populations with extended follow-up periods to assess knowledge retention and generalizability.

## 1. Introduction

Periodontitis, a severe form of gum disease that destroys the supporting structures of the teeth, represents a silent but significant global health crisis [1]. Affecting nearly 11% of all adults worldwide [2], this chronic inflammatory condition extends far beyond the oral cavity, with mounting evidence linking it to systemic diseases such as diabetes, cardiovascular disorders, and adverse pregnancy outcomes [3]. The global burden of periodontal disease is particularly pronounced in low- and middle-income countries, where prevalence rates exceed 40% in certain adult populations, and access to preventive care remains severely limited [4,5]. Recent epidemiological analyses indicate that severe periodontitis is the sixth most prevalent health condition globally, affecting approximately 796 million people and contributing substantially to years lived with disability [6,7]. Despite its profound impact on quality of life and its status as the primary cause of tooth loss in adults, periodontitis remains alarmingly underprioritized in public health agendas [8,9]. The disease progresses insidiously, often painlessly, meaning that by the time individuals seek treatment, irreversible damage has already occurred [10,11]. This trajectory of silent destruction underscores a critical imperative: shifting the paradigm from late-stage intervention toward early, proactive prevention.

The window for establishing effective prevention habits is arguably most critical during emerging adulthood, a developmental stage characterized by increasing autonomy and the crystallization of lifelong health behaviors. University students, typically aged 18 to 29, occupy a unique intersection of vulnerability and opportunity [12]. While they may perceive themselves as invulnerable to chronic diseases [13], this period is precisely when the foundational habits of oral hygiene—or neglect thereof—begin to manifest as clinical consequences in later decades [14,15]. Contemporary health behavior research has demonstrated that interventions targeting university students can yield substantial long-term benefits, as this population demonstrates both cognitive receptivity to health messaging and the developing autonomy to implement behavioral changes independently [16,17]. The university setting thus presents an unparalleled opportunity for targeted educational interventions. By equipping young adults with accurate knowledge about periodontitis prevention, risk factors such as smoking and diabetes [18], and the significance of early warning signs like bleeding gums [19], we can potentially alter the disease trajectory for an entire generation before pathological processes become entrenched.

Within the Ecuadorian context, this need is both urgent and, until now, entirely unmet. Our systematic review of serious games developed for the Ecuadorian healthcare sector between 2018 and 2024 [20], revealed a stark and concerning gap. While innovative digital tools have been successfully deployed for sexual health education in adolescents with intellectual disabilities, for physical and cognitive rehabilitation in children, and even for promoting healthy lifestyles through escape room methodologies, not a single serious game has been designed to address periodontitis literacy within the country. This absence is particularly striking given Ecuador’s burden of oral disease and the demonstrated efficacy of gamified interventions in other health domains. The nation has pioneered culturally adapted solutions for autism spectrum disorder and ADHD, yet oral health—a cornerstone of overall well-being—remains conspicuously absent from the serious games landscape.

The pedagogical justification for gamified learning tools in health education is well-established in contemporary educational research. Serious games leverage multiple mechanisms that enhance learning outcomes compared to traditional didactic instruction: they provide immediate corrective feedback, which strengthens memory consolidation through the testing effect [21,22]; they increase learner engagement through intrinsic motivation and autonomy satisfaction, as articulated in self-determination theory [23]; and they enable adaptive difficulty progression that maintains optimal challenge levels for individual learners [24,25]. Meta-analyses of digital health interventions have demonstrated that gamified approaches produce moderate to large effect sizes for knowledge acquisition, with particularly pronounced advantages for populations possessing high digital literacy [26,27]. Within the specific domain of oral health education, systematic reviews have identified interactivity, feedback immediacy, and motivational design as critical components distinguishing effective from ineffective interventions [28,29].

This confluence of epidemiological urgency, technological readiness, and pedagogical opportunity—coupled with the complete absence of oral health serious games in Ecuador—provides the precise context in which the present study positions itself. We focus on a highly specific yet strategically significant population: tenth-semester Software Engineering students at the University of Guayaquil. These individuals stand at the precipice of professional life, poised to enter Ecuador’s burgeoning technology sector. Their age profile, predominantly ranging from 21 to 32 years, places them squarely within the demographic where early periodontitis prevention should commence in earnest. Yet, as our pre-test data unequivocally demonstrate, baseline knowledge of gum disease among these students hovers around a mere 20%, indicating that conventional health education channels have failed to reach them. Paradoxically, these same students possess uniformly high digital literacy and are intimately familiar with interactive technologies—a combination of ignorance and capability that transforms them from a passive target population into an ideal testing ground for innovation.

The selection of Software Engineering students is thus neither arbitrary nor merely convenient. As future creators of technology, their engagement with the GUM game serves a dual purpose: they are simultaneous learners acquiring vital health literacy and critical evaluators whose feedback can inform the next generation of educational tools. By demonstrating the efficacy of a gamified, adaptive learning environment within this technologically sophisticated cohort, we not only address an immediate educational deficit but also cultivate a cadre of professionals who may themselves become advocates and developers of digital health interventions. In a nation where serious games for periodontitis are nonexistent, the University of Guayaquil’s tenth-semester cohort represents both the starting line and the proving ground—a deliberate first step toward closing a gap that has persisted for far too long in Ecuadorian public health.

The remaining sections of the paper are structured as follows: Section 2 presents the research questions and hypotheses guiding the comparative evaluation of the GUM game against traditional instruction. Section 3 reviews related work in digital health interventions and gamified learning for oral health education, identifying the distinct gap that this study addresses. Section 4 provides a comprehensive description of the GUM game’s design, including its welcome screen, question interface, feedback mechanisms, and both individual and group reporting features. Section 5 details the experimental design, participant recruitment, randomization procedure, and the structured pre-test/intervention/post-test protocol. Section 6 reports the quantitative results, encompassing baseline equivalence, post-intervention performance comparisons, statistical significance testing, and error reduction analysis. Section 7 discusses the implications of findings, study limitations, and considerations for broader application. Finally, Section 8 concludes the paper with a summary of contributions and outlines directions for future research, including longitudinal retention studies and cross-population validation.

## 2. Research Questions and Hypothesis

This study was driven by a central research question: Does the interactive, gamified learning environment of the GUM game lead to a greater improvement in periodontitis literacy among tenth-semester Software Engineering students at the University of Guayaquil compared to traditional lecture-based instruction? To address this question empirically, we formulated two directional hypotheses grounded in the pedagogical principles of serious game design and health education research.

**Hypothesis 1** **(H1).** *The use of the GUM game significantly improves periodontitis literacy, resulting in higher post-test scores and a greater reduction of misconceptions compared to pre-test levels among participants in the intervention group.*

**Hypothesis 2** **(H2).** *Traditional lecture-based instruction does not lead to a significant improvement in periodontitis literacy, with post-test scores and misconception rates remaining statistically unchanged or showing only minimal improvement compared to the interactive game-based approach.*

These hypotheses will be tested through a controlled experimental design, comparing pre- and post-intervention knowledge assessments between an intervention group using GUM and a control group receiving traditional instruction. The results will be analyzed to determine whether the gamified, adaptive, and feedback-driven pedagogy of GUM offers a measurable advantage in periodontitis literacy education for our specific population. We will answer our research question and examine whether our hypotheses are true in Section 6.

## 3. Related Work

Table 1 presents a diverse landscape of digital interventions aimed at improving periodontitis literacy, spanning various target populations, pedagogical strategies, and technological platforms. It summarizes recent innovations that have demonstrated the efficacy of game-based strategies across diverse populations and formats. Collectively, these works affirm that interactivity, immediate feedback, and motivational design elements can meaningfully amplify learning outcomes across age groups and delivery modalities.

Yet, the very richness of this landscape throws into sharp relief the gap that GUM is designed to fill. No serious game to date has targeted periodontitis literacy specifically within the emerging adult population—a developmental window critical for crystallizing lifelong preventive behaviors—nor has any intervention been tailored to the unique profile of university-level Software Engineering students in Latin America. More fundamentally, the existing interventions diverge from GUM in their pedagogical architecture and scope of autonomy. Kumar et al. [30], Peter et al. [31], and Santhosh et al. [32] all relied on facilitator-led, classroom-based delivery incorporating physical demonstrations, tactile materials, or live quizzes; these are inherently resource-intensive and lack scalability. Chang et al. [33] embedded metacognitive scaffolding within a social 2D environment, yet their approach retained facilitator-led feedback and emphasized collaborative reflection over individual knowledge mastery. Morariu et al. [34] and Chang et al. [35] prioritized longitudinal behavior changes through daily habit support, employing reminders and push notifications rather than immediate, concentrated knowledge acquisition.

In contrast, GUM is a fully digital, self-directed serious game that operates without any facilitator or clinical component. It delivers a concentrated, single-session learning experience driven by AI-informed adaptive feedback, real-time scoring, and granular individual performance analytics. Its pedagogical engine is not habiting formation over weeks, but iterative misconception correction within minutes—transforming each question, whether answered correctly or incorrectly, into an elaborated instructional event. By embedding comprehensive summative reports and group statistics, GUM also fosters metacognitive reflection and social comparison within a cohort, features absent from comparator tools. Thus, while prior works establish gamification as a viable vehicle for oral health education, GUM pioneers a distinct, scalable paradigm: autonomous, feedback-dense, analytics-rich serious gameplay purpose-built to close the periodontitis literacy gap in precisely the population where inattention to gum health portends the greatest future burden.

**Table 1:** Works that use digital interventions for improving periodontitis literacy and differences with our study.

Authors	Population	Goal	Approach	Main Findings	Differences with the GUM Game
Kumar et al. (2022) [30]	100 adolescents (12–15 years) in India	To evaluate the effect of an interactive game-based visual performance (IGVP) technique vs. conventional oral health education (OHE) talk on plaque control, gingival health, and oral hygiene knowledge/practices.	IGVP (animated video + Kahoot quiz + brushing demonstration) vs. Conventional OHE talk	IGVP group showed significant reductions in gingival (58.7%) and plaque scores (63.4%) and greater knowledge gain (22.4%) compared to the control group (2.8%, 0.7%, and 7.8% respectively).	Unlike Kumar et al.’s classroom-based IGVP technique—which combined animated videos, live Kahoot quizzes, and physical brushing demonstrations for adolescents—the GUM game is a fully digital, self-directed serious game targeting university students, featuring AI-informed adaptive feedback, real-time scoring, and individual performance analytics without any facilitator or clinical component.
Peter et al. (2025) [31]	35 schoolchildren (9–12 years) in Tamil Nadu, India	To evaluate the effectiveness of game-based vs. conventional oral health education in improving knowledge, attitude, practice (KAP), and oral hygiene status.	Game-based (match-the-following, sorting activity) vs. Conventional OHE (demonstration, instruction)	Game-based group showed significant improvement in KAP scores and oral hygiene (77.8% achieved “Good” OHIS) vs. control group (23.5%). Conventional group showed a non-significant decline.	Peter et al.’s intervention used low-tech, classroom-based games like match-the-following and food sorting activities, delivered in person with brushing demonstrations for children aged 9–12. GUM targets individual knowledge mastery through software, while Peter emphasizes hands-on group learning without digital components.
Santhosh et al. (2024) [32]	195 adolescents (12–15 years) in Belagavi, India	To assess the effectiveness of a Jigsaw Puzzle-assisted Visual Reinforcement (JPVR) technique vs. conventional OHE and video demonstration on toothbrushing knowledge, practices, and clinical parameters.	JPVR (jigsaw puzzle + visual stickers) vs. Video demonstration vs. Conventional OHE	JPVR group showed significantly higher knowledge and practice scores, and greater reduction in plaque and gingival scores at 3 months compared to other groups.	Santhosh et al.’s JPVR technique is a low-tech, collaborative classroom intervention where adolescents assemble physical jigsaw puzzles and use visual sticker reinforcements, guided by facilitators. GUM emphasizes autonomous knowledge mastery through software, while JPVR leverages tactile group problem-solving and extrinsic rewards without any digital or adaptive components.
Morariu et al. (2025) [34]	18 adult patients (periodontitis focus) in Romania	To develop and pilot-test a mobile app (PerioSupportPro) for improving daily oral hygiene adherence and periodontitis management.	Mobile Health App (mHealth) with gamification, reminders, videos	App was well-received; reminders, interface, and videos were most valued. Motivation section showed good internal consistency (α=0.784). High overall satisfaction correlated with reminders and motivational features.	The GUM game is a one-session, web-based serious game that uses adaptive AI-informed quizzes and performance analytics to improve periodontitis literacy among university students. In contrast, Morariu et al.’s PerioSupportPro is a habit-support mHealth app for long-term daily use, featuring reminders, video tutorials, badges, and progress tracking to sustain oral hygiene adherence in adult periodontitis patients. GUM focuses on immediate knowledge gain through interactive assessment, while PerioSupportPro prioritizes longitudinal behavior change and motivational reinforcement via push notifications and rewards.
Chang et al. (2025) [33]	48 university students (20–24 years) in Taiwan	To evaluate a WSQ (Watch–Summarize–Question) gamified learning approach vs. video-based learning on flossing knowledge, motivation, and skill performance.	WSQ Gamified Learning (via Gather.town) vs. Video-Based Learning	WSQ group had significantly higher learning achievement in flossing knowledge. No significant differences in motivation or flossing skill performance, though WSQ group scored slightly higher in skills.	The GUM game is a self-directed serious game for university students that uses AI-informed adaptive feedback, scoring, and individual analytics to improve periodontitis literacy, with no facilitator required. In contrast, Chang et al.’s WSQ gamified approach targets younger adults via Gather.town, embedding structured Watch-Summarize-Question metacognitive prompts and facilitator-led feedback to teach flossing skills, but found no significant motivational or skill performance advantage over video learning. GUM emphasizes autonomous mastery and misconception correction, while Chang prioritizes scaffolded reflection within a social 2D environment.
Chang et al. (2024) [35]	30 school-aged children (5–12 years) and their parents in Taiwan	To develop and evaluate an educational chatbot (COSC) to promote oral self-care using the Behavior Change Wheel (BCW) framework.	Gamified Chatbot (LINE-based) with BCW design (capability, motivation, opportunity)	Chatbot showed good usability (mean CUQ score 79.91) and high likeability (mean 4.32/5). Most valued features: guided toothbrushing and quizzes/challenges.	Chang et al.’s COSC is a theory-driven, menu-based LINE chatbot for Taiwanese children, designed using the Behavior Change Wheel to build oral self-care habits via structured education, gamified quizzes, and guided brushing videos. GUM focuses on immediate periodontitis literacy through assessment, while COSC emphasizes longitudinal behavior change, parental involvement, and accessibility via a familiar social messaging platform.

## 4. Game Design

The game proposed in this work is called GUM, an acronym of “Gum Understanding Mission”. The game is available at [36] and consists of a series of multiple-choice questions focused on health literacy. More specifically, it is focused on the pathology of periodontitis, designed as a serious game for last-semester software engineering students at the University of Guayaquil. Its pedagogical design leverages game mechanics—such as timed challenges, a scoring system, lives, and immediate corrective feedback—to create an engaging and reflective learning experience. The structure progresses from an introductory welcome screen that establishes relevance and stakes, through iterative assessment screens with real-time feedback, to comprehensive summative reports. These final reports provide both individual performance analytics and group statistics, facilitating self-regulated learning, metacognitive review, and social comparison within an educational cohort.

### 4.1. Welcome Screen

**Figure 1:**
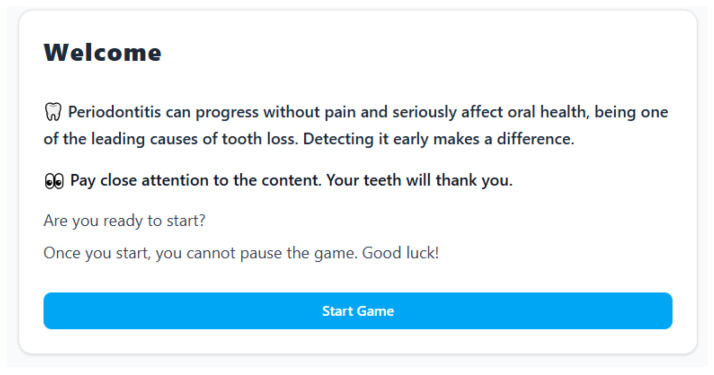
Welcome screen in the GUM game.

The welcome screen of the “GUM: Gum Understanding Mission” serious game (depicted in Figure 1) serves as a critical pedagogical gateway, skillfully employing design elements to frame the player’s mindset and establish the game’s educational purpose. Pedagogically, it functions not merely as a menu but as an anticipatory set, a foundational educational strategy used to activate prior knowledge and prime learners for the incoming material. The stark, direct statements about periodontitis—noting its painless progression and severe consequences—immediately construct a “need to know.” This addresses a key challenge in adult and professional education: motivation. By presenting the stakes (“seriously affect oral health,” “leading cause of tooth loss”) upfront, the design creates cognitive dissonance for the player, likely a software engineering student who may not perceive oral health as immediately relevant. It answers the unspoken question, “Why should I care?” before the game begins, thereby enhancing intrinsic motivation to engage with the content that follows.

The narrative tone and instructional cues are carefully crafted pedagogical tools. The phrase “Pay close attention to the content. Your teeth will thank you” employs a mix of direct instruction and subtle, relatable personification. It transforms the learning objective from an abstract requirement (“improve literacy”) into a personal, future-oriented benefit. This leverages the pedagogical principle of relevance, crucial for engaging last-semester students who are likely focused on immediate career goals. The warning that the game cannot be paused serves a dual pedagogical design function. First, it mimics the high-stakes, immersive environments familiar in software engineering (like deployment or testing phases), creating a familiar frame of challenge. Second, it promotes focused, uninterrupted cognitive engagement, reducing distractions and encouraging deep processing of the game’s information, which is essential for converting short-term interaction into long-term knowledge retention.

Finally, the clean, uncluttered layout with the prominent [Start Game] button minimizes extraneous cognitive load, a core tenet of multimedia learning theory. All attention is funneled toward the key message and the singular action of commencement. In summary, the welcome screen is a masterclass in concise instructional design: it establishes relevance, creates stakes, commands focused attention, and builds credibility, all within a user interface that respects the cognitive style and professional context of its target learner—the software engineering student.

### 4.2. Questions Screen

**Figure 2:**
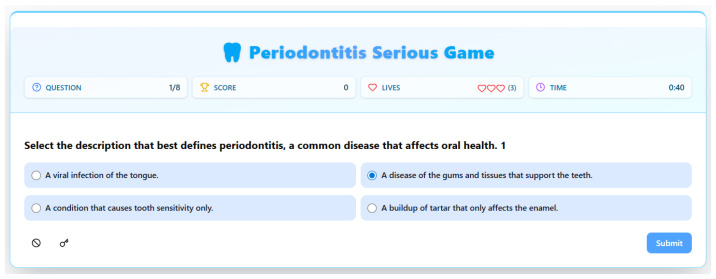
Questions screen in the GUM game.

The design of this gameplay screen (Figure 2) exemplifies a direct and efficient pedagogical approach, meticulously structured to assess and reinforce foundational knowledge within an engaging, game-based framework. The screen is immediately recognizable as an assessment interface, a digital quiz that leverages the motivational mechanics of gaming to transform a learning checkpoint into an active challenge. The prominent headers for “Question”, “Score”, “Lives”, and “Time” are not merely decorative; they are core pedagogical tools. They establish clear rules and provide continuous, formative feedback, key principles in both educational design and game-based learning. The numerical indicators (1/8, 0, 0, 0:40) create a narrative of progression and limitation. The question counter breaks the learning objective into manageable chunks, reducing potential cognitive overload. The score and lives systems introduce stakes and immediate consequences for performance, fostering a sense of accountability and challenge. To maintain engagement and introduce consequence-driven decision-making, players begin with three lives, and one life is deducted for each incorrect response. This penalty is immediately reflected in the interface, reinforcing the seriousness of the task while maintaining a supportive tone through encouraging feedback messages. The system is designed not to end gameplay prematurely, but to foster a sense of accountability and focus, thereby enhancing the learning experience. Most critically, the timer injects a layer of controlled pressure, simulating the need for quick recall and decision-making, which can enhance focus and mimic real-world scenarios where timely identification of health information might be crucial.

Pedagogically, the construction of the multiple-choice question itself is carefully designed for clarity and to diagnose specific misconceptions. The stem is unambiguous: “Select the description that best defines periodontitis.” This language cues the player to look for the most comprehensive and accurate answer, moving beyond partial truths. The distractors are not random but are crafted to represent common misunderstandings or oversimplifications of oral health issues. Options like “A viral infection of the tongue” and “A condition that causes tooth sensitivity only” target fundamental errors in identifying the disease’s nature and scope. The correct answer, “A disease of the gums and tissues that support the teeth,” is stated with precise, clinical terminology, reinforcing the exact literacy the game aims to build. This structure follows best practices in assessment design by ensuring that correct answers require genuine understanding rather than guesswork from implausible options.

Furthermore, the entire screen layout minimizes extraneous cognitive load, a central tenet of multimedia learning theory. The visual hierarchy is clear: the question is the focal point, with the interactive answer choices centered below. The game-state information (score, time) is persistently visible but relegated to the top corners, allowing monitoring without distraction. The stark contrast and simple interface ensure the player’s cognitive resources are devoted almost entirely to the content of the question and the logic of their selection. The [Submit] button acts as a definitive commitment mechanism, requiring active confirmation of the choice and marking a clear transition from thinking to feedback, which would presumably follow. In essence, this screen is a potent fusion of instructional design and game mechanics. It uses the familiar, engaging format of a timed quiz—a structure well-understood by students—to deliver targeted, diagnostic assessment, immediately applying the foundational knowledge introduced in the welcome screen and setting the stage for iterative learning through subsequent questions and challenges.

### 4.3. Feedback Screen

**Figure 3:**
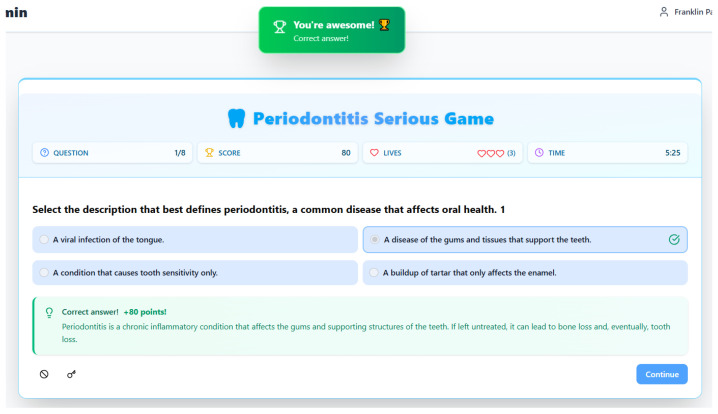
Feedback screen that follows the player’s correct answer in the GUM game.

Building upon the pedagogical framework established in the initial assessment screen, the game’s paired feedback screens for correct and incorrect answers form a sophisticated instructional system designed to guide learning through immediate reinforcement and corrective explanation. These screens move beyond simple binary feedback, leveraging the motivational core of game mechanics to deepen conceptual understanding. The positive feedback screen (Figure 3), triggered by a correct answer, employs celebratory language (“You’re awesome!”) and a points reward (“+80 points!”) to provide powerful positive reinforcement. This aligns with behavioral learning principles, directly linking the successful retrieval of knowledge with a rewarding emotional and visual payoff, thereby increasing the likelihood of future engagement and recall. Crucially, it follows this with an elaborated feedback paragraph that expands on the correct answer with precise clinical terminology (“chronic inflammatory condition”) and reiterates the serious consequences (“bone loss… tooth loss”). This transforms a correct guess into a confirmed learning moment, solidifying the accurate mental model.

**Figure 4:**
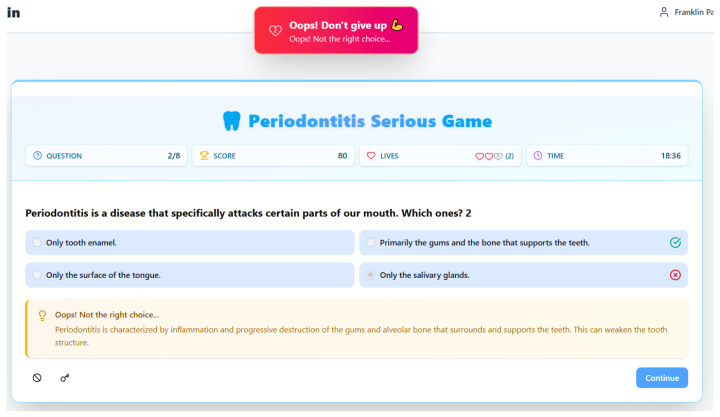
Feedback screen that follows the player’s incorrect answer in the GUM game.

Conversely, the negative feedback screen for an incorrect answer (Figure 4) is pedagogically designed not to punish, but to teach and encourage resilience. The tone is supportive (“Oops! Don’t give up”), mitigating frustration and maintaining motivation—a critical aspect of creating a safe learning environment where errors are part of the process. Like its positive counterpart, it provides immediate and elaborated feedback, stating “Oops! Not the right choice…” and offering a clear, corrective explanation. The explanatory text (“inflammation and progressive destruction of the gums and alveolar bone”) serves a vital corrective function by explicitly dismantling the player’s misconception and replacing it with the accurate, detailed definition. This is a direct application of the “testing effect” and corrective feedback, where an incorrect attempt, when followed by the right information, can lead to stronger long-term memory formation than passive study.

Both screens share a consistent design philosophy that maintains the game-state context to foster metacognition. They persistently display the updated “Score”, “Lives”, and “Time”, with the lives counter visually diminishing on an incorrect answer (from 3 to 2), introducing tangible stakes that underscore the importance of accurate knowledge without being terminally punitive. The time bonus on a correct answer (increasing from a starting 0:40 to 5:25) and the substantial time shown after an incorrect answer (18:36) likely incentivizes both speed and accuracy as complementary skills. Importantly, both screens leave the original question and answer choices visible, allowing the player to review the problem space in the illuminating context of the feedback. The solitary [Continue] button on each screen gives the learner agency over the pacing, ensuring they can fully process the explanatory feedback before proactively moving forward. In essence, this two-pronged feedback system is the engine of the game’s pedagogy. It ensures that every interaction—whether a success or a mistake—becomes a productive learning event, using the engaging framework of game mechanics (points, lives, time) to deliver immediate, elaborated feedback that either reinforces precise knowledge or effectively corrects misconceptions, thereby systematically building the player’s periodontitis literacy.

### 4.4. Concluding Screen

**Figure 5:**
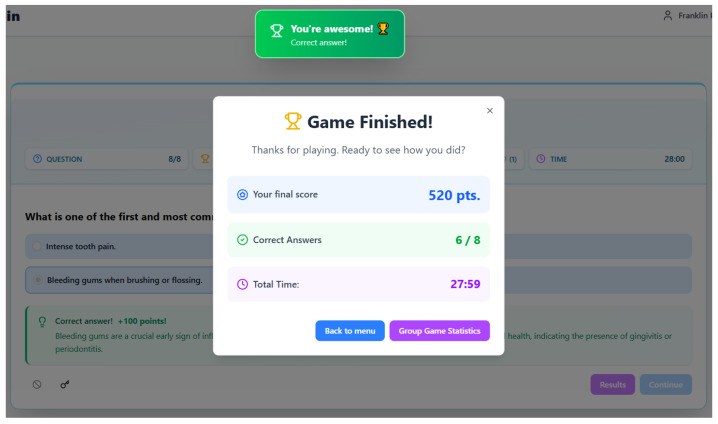
Concluding screen of the GUM game.

The concluding screen of the “GUM” serious game (Figure 5) represents the pedagogical culmination of the experience, masterfully designed to provide closure, summarize achievement, and deliver a final, resonant learning point. It transitions the player from the iterative challenge-feedback loop of the gameplay into a reflective and summative space. The screen opens with a celebratory reaffirmation (“You’re awesome!”), extending the positive reinforcement strategy used throughout to frame the entire experience as a success, regardless of the specific score. This is crucial for maintaining motivation and a positive association with the learning content. The declaration “Game Finished!” and the prompt “Ready to see how you did?” formally mark the end of the challenge phase and initiate a metacognitive review, inviting the player to reflect on their performance—a key step in consolidating learning.

Pedagogically, the screen merges summative assessment with one last formative touch. The presentation of the final score (520 pts.), correct answers (6/8), and total time (27:59) provides a comprehensive performance summary. These metrics cater to different reflective dimensions: the score offers a gamified measure of overall success integrating speed and accuracy; the correct-answers ratio gives a straightforward competency percentage; and the total time contextualizes the engagement. This data allows the learner to self-assess their mastery level and the effort invested. Most strikingly, the design includes a final, seemingly integrated multiple-choice question (“What is one of the first and most common issues?”). This is a sophisticated pedagogical device. By presenting and immediately answering it (“Bleeding gums when brushing or flossing… +100 points!”), the game performs a final knowledge reinforcement. It emphasizes a single, critical, and actionable takeaway—the significance of bleeding gums as an early warning sign—ensuring that even if a player forgets detailed pathology, they retain a practical piece of literacy that can prompt real-world action. This transforms the summary screen from a mere scoreboard into a final, memorable teaching moment.

Furthermore, the navigation options ([Back to menu] and [Group Game Statistics]) serve important extended pedagogical functions. They return agency to the player, allowing them to exit or to engage in social comparison. The “Group Game Statistics” option is particularly potent, leveraging the collaborative context of a classroom. It can foster discussion, normalize difficulty, and create a community of learning around the topic, moving the experience from individual gameplay to a shared academic reference point. In essence, this ending screen is designed as a cohesive epilogue. It celebrates effort, quantifies learning, implants a key practical message, and provides pathways for further exploration or community integration. It ensures the game’s educational impact resonates beyond the final click, aiming to leave the software engineering student not just with a score, but with a durable, useful piece of health literacy.

### 4.5. Group Statistics Screen

**Figure 6:**
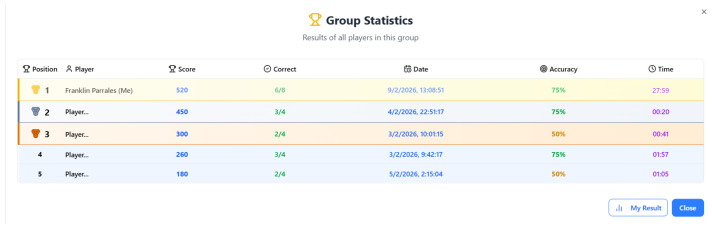
The Group Statistics screen of the GUM game.

The Group Statistics screen (Figure 6) represents a sophisticated pedagogical extension of the serious game, moving beyond individual knowledge assessment to leverage social learning and metacognitive reflection within a cohort. By presenting a ranked leaderboard of all players of the group, the design taps into powerful motivational drivers familiar in both gaming and educational environments: comparison, competition, and community. This screen transforms the learning experience from a private activity into a shared, social endeavor. For the player, seeing their own entry highlighted as “Franklin Parrales (Me)” at the top provides a strong sense of accomplishment and positive reinforcement, validating their effort and mastery. However, the pedagogy is nuanced; it is not purely about winning. The inclusion of multiple metrics—Score, Correct, Accuracy, and Time—shifts the focus from a single dimension of success to a multifaceted view of performance. A player can see, for instance, that while they scored highest (520), their accuracy (75%) is matched by Player 2, who achieved it in a dramatically shorter time (00:20). This complexity encourages deeper reflection on the trade-offs between speed and thoroughness, or between guessing and deliberate reasoning, fostering higher-order thinking about one’s own learning process.

Pedagogically, the data-rich table serves as a powerful tool for normative and formative self-assessment. The “Accuracy” column is particularly instructive, providing a clear, percentage-based measure of conceptual understanding separate from the game’s points system, which may reward speed bonuses. This allows learners to identify whether their core knowledge is strong (high accuracy) or if their score was inflated by other factors. The presence of other players with varying results (e.g., 50% vs. 75% accuracy) normalizes a range of outcomes, reducing potential anxiety by showing that struggle is part of the learning process. The inclusion of Date and Time stamps contextualizes performance, potentially revealing patterns (e.g., performance at different times of day) or simply lending authenticity and a sense of an ongoing, active learning community. This communal data set can demystify the learning objectives, making the expected performance range transparent and providing concrete benchmarks for self-improvement.

Furthermore, the design choices promote agency and focused reflection. The two buttons, [My Result] and [Close], offer clear, simple pathways. “My Result” could allow the player to isolate and review their personal performance data in detail, facilitating private self-assessment without social comparison—a crucial option for reducing potential negative effects of ranking. “Close” provides a clean exit, respecting the player’s autonomy. Presenting this screen after the final score summary creates a coherent narrative arc: from individual performance review to social contextualization. In a classroom setting of software engineering students, this screen could be a springboard for instructor-led discussions about common misconceptions (gleaned from low-accuracy trends) or the relationship between time pressure and accuracy. Thus, the Group Statistics screen elevates the game’s pedagogy by embedding individual learning within a social framework, encouraging analytical reflection on performance metrics, and fostering a sense of collective endeavor that can enhance engagement and deepen the educational impact of the entire “GUM” experience.

### 4.6. Individual Game Report

**Figure 7:**
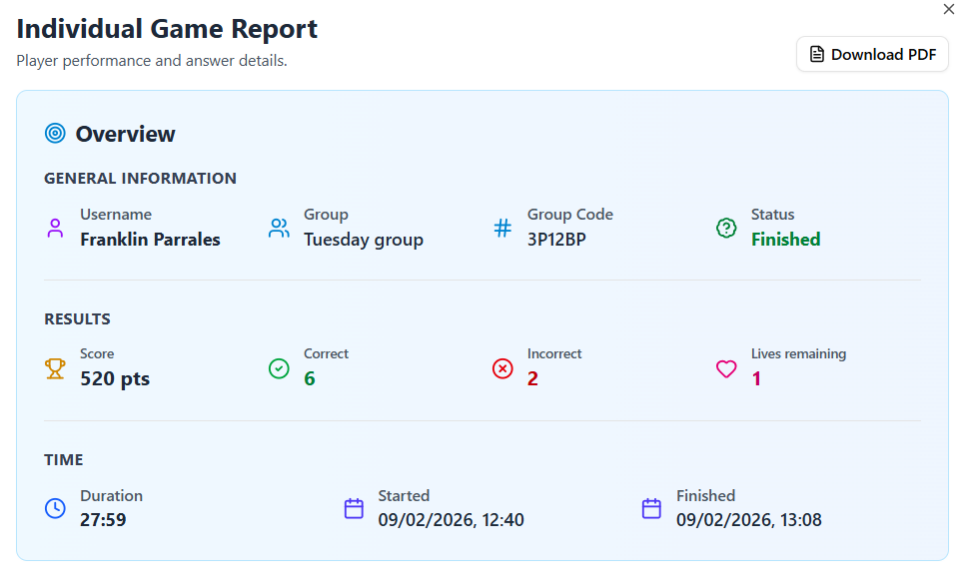
The initial “Overview” part of the Individual Game Report of the GUM game. In this figure, only partial details of the report are shown. The complete content of the individual report (including the answer options, feedback, and response times) can be viewed by scrolling down the report.

**Figure 8:**
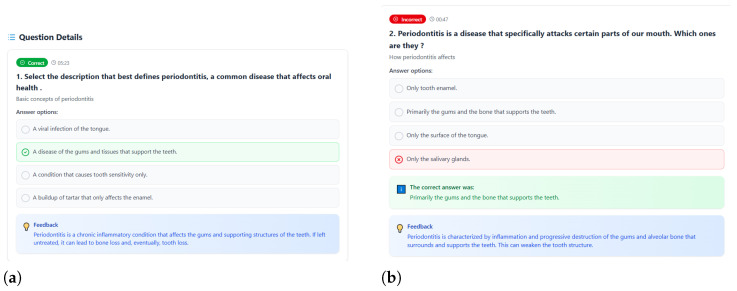
Question details for (**a**) correct and (**b**) incorrect answered questions of the Individual Game Report of the GUM game. In this figure, only partial details of correct and incorrect answers are shown. The complete content of the individual report (including the remaining questions, answer options, feedback, and response times) can be viewed by scrolling down the report.

The Individual Game Report (Figure 7 and Figure 8) represent the deepest layer of pedagogical design in the serious game, shifting from the immediate engagement of gameplay and social comparison to a structured, analytical space for self-regulated learning and metacognitive review. This detailed post-game analysis is where the experience transitions from assessment to genuine, personalized study. The initial “Overview” part of the report (Figure 7) establishes the learner’s performance within a formal, almost academic, context. By presenting “General information” (Username, Group, Code, Status) alongside “Results” and “Time” data, it frames the gameplay as a documented learning event. This design choice subtly elevates the activity from a casual game to a legitimate educational exercise, appealing to the software engineering students’ familiarity with data logs and performance reports. The inclusion of precise timestamps (“Started,” “Finished”) and total duration not only provides context but also encourages reflection on time management and sustained focus during the learning task.

The core pedagogical power lies in the “Question Details” section, which functions as a personalized learning dashboard. By breaking down performance question-by-question, the design facilitates targeted review and remediation—a critical principle in mastery learning. The labeling of each question with its outcome (Correct in Figure 8a or Incorrect in Figure 8b) and the time taken to answer (e.g., “05:23,” “00:47”) provides profound insight into the player’s cognitive process. A long time on a correct answer might indicate careful deliberation or uncertainty, while a very fast incorrect answer suggests a strong but wrong intuition or a guess. This temporal data pushes the learner to reflect not just on what they knew, but how they knew it. The restatement of each question, its answer options, and the categorization (e.g., “Basic concepts of periodontitis,” “How periodontitis affects”) allows the player to re-contextualize the challenge. Crucially, the screen always provides the correct answer and the full Feedback text for every item, regardless of whether the player got it right or wrong. This ensures that even correctly answered questions are reinforced with the elaborated explanation, and incorrect ones receive the essential corrective information needed to rebuild understanding.

Furthermore, the interactive design elements promote active, learner-driven review. The buttons (Correct, Time, Close) at the bottom of the detailed question view suggest the ability to sort or filter the report, perhaps to quickly review all mistakes or analyze time patterns. This grants agency to the learner, allowing them to direct their review based on their own identified needs—a key aspect of fostering self-regulated learning skills. The report essentially acts as a “debugging” tool for the player’s own knowledge base. For a software engineering student, this mirrors the process of reviewing a code log or test results: identifying specific errors, understanding why they happened (via the feedback), and solidifying correct procedures. In this way, the Individual Game Report screens complete the pedagogical circuit. They transform the aggregated score from the final screen into actionable intelligence, guiding the learner to a precise understanding of their strengths and weaknesses, and ensuring the game’s ultimate goal—improving periodontitis literacy—is achieved not just through play, but through thoughtful, data-informed reflection.

## 5. Experimental Design

**Figure 9:**
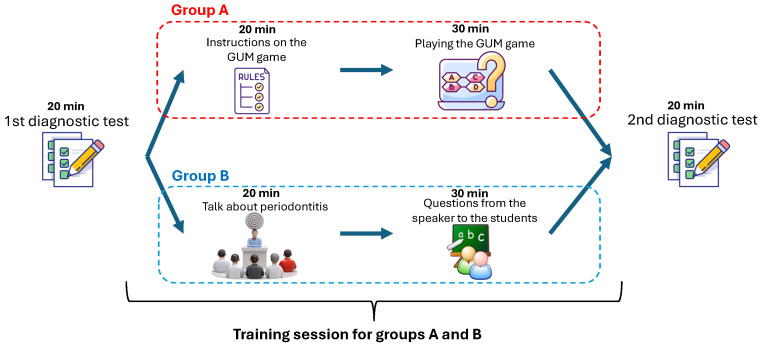
Experimental design to measure the improvement in the gum disease literacy.

This study was structured as a controlled, comparative intervention aimed at rigorously evaluating the pedagogical impact of the GUM game (available in [36]) on periodontitis (gum disease) literacy. The core objective was to determine whether an interactive digital game could serve as a more effective educational tool than conventional didactic instruction. To this end, a pre-test/post-test experimental framework was adopted, allowing for a direct quantitative comparison of knowledge acquisition between two distinct instructional modalities. The entire experimental sequence—from participant recruitment through final assessment—was meticulously planned and standardized to ensure internal validity and reproducibility. A visual summary of this end-to-end protocol is provided in Figure 9, which delineates the parallel tracks for the intervention and control groups across all phases of the study. A dedicated facilitator oversaw all sessions to guarantee consistent delivery of instructions, proper management of materials, and strict compliance with the established research protocol.

### 5.1. Participant Profile and Sampling

The participant pool was deliberately drawn from a specific academic cohort to control for variables such as general academic maturity and familiarity with technology. A total of 50 undergraduate students volunteered to participate. Verbal informed consent used is available in Appendix A. These students were all enrolled in their final academic term (10th semester) of the Software Engineering degree program at the University of Guayaquil during the second semester of the 2025 academic year. This sample constituted 78.12% of the total population of 64 students registered for that semester, indicating a high level of voluntary participation. The cohort was predominantly young adults, with ages ranging from 21 to 32 years. The gender distribution was 78% male (n = 39) and 22% female (n = 11), a ratio reflective of the typical enrollment patterns in software engineering programs. A key, and intentional, characteristic of this sample was their substantial prior exposure to information and communication technologies (ICT). This ensured a uniformly high baseline of digital literacy, thereby minimizing potential confounding effects stemming from disparities in digital comfort or skill when interacting with the GUM game interface. Exclusion criteria included prior formal dental education, current or previous periodontitis diagnosis, and participation in any oral health education program within the preceding 12 months.

### 5.2. Randomization Procedure

To mitigate selection bias and ensure group equivalence at baseline, the 50 volunteers were randomly assigned to one of two conditions using a rigorous randomization protocol. A computer-generated random number sequence was created using Research Randomizer software (available in https://www.randomizer.org/ (accessed on 10 January 2026)) with a 1:1 allocation ratio. The randomization sequence was generated by an independent researcher not involved in participant recruitment or data collection. Allocation concealment was maintained through the use of sequentially numbered, opaque, sealed envelopes prepared prior to participant enrollment. Each envelope contained a group assignment card (either “Group A” for GUM game intervention or “Group B” for traditional lecture). Upon confirmation of eligibility and completion of informed consent, each participant opened the next envelope in the sequence in the presence of the facilitator, revealing their group assignment. This process resulted in the formation of two groups, each comprising 25 participants. Group A was designated as the experimental or intervention group, with their learning experience centered entirely around the GUM game. Group B was designated as the control group, receiving instruction through a traditional, instructor-led lecture format, serving as the benchmark against which the game’s effectiveness was measured.

### 5.3. Assessment Instrument: Development and Psychometric Validation

The knowledge assessment instrument used for both pre-test and post-test measurements was developed through a multi-stage validation process to ensure its psychometric adequacy for measuring periodontitis literacy.

#### 5.3.1. Instrument Development

The assessment consisted of five carefully constructed multiple-choice questions, each presenting four plausible answer options with only one correct response. It is available in Appendix B. The content domains covered essential topics in gum disease literacy, including: (1) etiology and primary risk factors (e.g., smoking, diabetes); (2) evidence-based preventive practices (e.g., effective brushing technique, flossing); (3) fundamental concepts related to disease screening and early signs (e.g., bleeding gums, pocket depth); (4) disease progression and complications; and (5) treatment modalities and professional care recommendations.

#### 5.3.2. Content Validity

Content validity was established through a systematic review by an expert panel comprising four dental professionals: two periodontists with clinical experience, one dental public health specialist, and one dental educator. Each expert independently rated each question for relevance, clarity, and representativeness using a 4-point Likert scale (1 = not relevant to 4 = highly relevant). The Content Validity Index (CVI) was calculated for each item (I-CVI) and for the overall instrument (S-CVI). Items achieving I-CVI scores below 0.78 were revised based on expert feedback. The final instrument demonstrated excellent content validity with S-CVI = 0.92, indicating strong agreement among experts regarding the instrument’s appropriateness for measuring periodontitis literacy.

#### 5.3.3. Pilot Testing and Item Analysis

Following expert review, the instrument was pilot-tested with 15 tenth-semester Software Engineering students who were not part of the main study sample (recruited from a parallel section of the same course). Pilot participants completed the assessment under identical conditions to those planned for the main study and provided feedback on question clarity and comprehensibility. Item analysis was conducted to evaluate:

- Item difficulty index (*p*-value): Calculated as the proportion of pilot participants answering each item correctly. Items with *p*-values between 0.35 and 0.65 were retained, indicating appropriate difficulty without floor or ceiling effects. Final items ranged from 0.38 to 0.62.
- Item discrimination index: Calculated using point-biserial correlation between item performance and total test score. All items demonstrated discrimination indices exceeding 0.30 (range: 0.32–0.48), indicating good ability to distinguish between high and low performers.
- Distractor analysis: All incorrect options attracted at least 5% of responses, confirming that distractors were functional and not obviously incorrect.

#### 5.3.4. Reliability Assessment

Internal consistency reliability was evaluated using the Kuder–Richardson Formula 20 (KR-20), appropriate for dichotomously scored items. The pilot sample yielded a KR-20 coefficient of 0.78, exceeding the acceptable threshold of 0.70 for research purposes and indicating adequate internal consistency.

Test-retest reliability was assessed by administering the same instrument to the pilot sample two weeks after initial completion, with no intervening periodontitis education. The intraclass correlation coefficient (ICC) for absolute agreement was 0.85 (95% CI: 0.72–0.93), demonstrating excellent temporal stability.

#### 5.3.5. Final Instrument

The validated five-item instrument was used for both pre-test and post-test assessments in the main study. A time limit of 20 min was established based on pilot testing, during which all pilot participants completed the assessment within 15 min, ensuring adequate time for careful response without time pressure confounds.

### 5.4. Experimental Procedure

The experiment was executed as a series of sequential, standardized stages, each designed to isolate and measure the effect of the independent variable (instructional method) on the dependent variable (periodontitis knowledge). The following narrative details each step, corresponding to the workflow in Figure 9.

#### 5.4.1. Baseline Knowledge Assessment (Pre-Test)

Prior to any intervention, both groups completed the identical validated diagnostic assessment (pre-test) in a proctored setting with a strict 20-min time limit. Assessments were administered simultaneously in separate classrooms to prevent contamination between conditions. Participants were instructed to answer all questions to the best of their ability and were not informed of their scores to prevent subsequent performance bias. Completed assessments were collected immediately and stored in sealed envelopes by the facilitator.

#### 5.4.2. Orientation and Training Intervention

Following the pre-test, each group engaged in a distinct, time-matched training session tailored to their assigned pedagogical method. This phase was critical for delivering the core educational content. Both interventions were delivered by the same facilitator (a trained health educator with experience in both traditional instruction and digital learning facilitation) following standardized protocols to ensure consistency.

- Group A (GUM Game Intervention): Participants in this group first received a concise, 20-min orientation session delivered in a computer laboratory equipped with individual workstations. The orientation familiarized them with the GUM game’s interface, navigation, core mechanics (scoring, lives, timer), and underlying educational objectives. A standardized script was used to ensure all participants received identical instructions. Following this introduction, participants entered a self-directed, interactive training phase. They actively engaged with the game’s curated question sets (8 questions in the main gameplay loop), which were thematically aligned with the pre-test content domains. A defining feature of this intervention was the game’s dynamic feedback system, which provided immediate, context-specific corrective or reinforcing messages after each response. Furthermore, the game’s adaptive logic subtly adjusted difficulty or focused on knowledge gaps based on the player’s ongoing performance, offering a personalized learning trajectory. Participants progressed through the game independently, with the facilitator available only for technical assistance (e.g., login issues, system errors) but not for content-related guidance.
- Group B (Traditional Instructor-Led Lecture): Participants in the control group attended a 20-min lecture delivered by the same facilitator in a standard classroom setting. The lecture was structured to cover the same conceptual territory as the GUM game, addressing periodontitis prevention, key detection methods, and essential oral health literacy principles. The delivery was expository, relying on verbal explanation and static slide presentations (10 slides), thereby emulating a conventional classroom learning environment. The lecture content was developed to mirror the information contained in the GUM game’s feedback explanations, ensuring content equivalence across conditions. Participants were permitted to take notes but were not provided with handouts.

#### 5.4.3. Applied Knowledge Reinforcement

To consolidate the information presented during the training phase, both groups participated in a subsequent 30-min applied practice session. This stage was designed to move beyond passive reception of information toward active engagement and clarification.

- Group A—GUM Game Continuation: Participants continued their immersion in the GUM game environment. During this extended practice period, they encountered additional question sets (8 new questions) and repeated exposure to previously answered items, reinforcing concepts through repetitive, feedback-driven interaction. The game continued to serve as the sole source of guidance and correction, with no facilitator intervention regarding content.
- Group B—Question-and-Answer Session: Participants transitioned to a moderated, interactive question-and-answer (Q&A) session. Led by the same facilitator, this forum allowed participants to seek clarifications, pose questions about the lecture material, and discuss concepts in a dialogic format. The facilitator provided verbal explanations and elaborations in real-time, following a standardized protocol that addressed anticipated questions while allowing for spontaneous inquiries. All participant questions were recorded for fidelity checking but not analyzed as part of the quantitative outcomes.

#### 5.4.4. Outcome Knowledge Assessment (Post-Test)

The final measurement phase occurred immediately after the reinforcement session (within 5 min of completion). Both groups completed a post-test assessment in their respective settings under identical proctored conditions. Crucially, this post-test was isomorphic to the pre-test; it used the same five validated multiple-choice questions, presented in the same order and format, under identical time constraints (20 min). This design feature was essential for ensuring that any measured change in score could be directly attributed to the learning intervention rather than to differences in test difficulty. To prevent response bias or strategic changes in test-taking behavior, participants were deliberately not informed of their scores on either the pre-test or the post-test at any point during the study. This blinding helped ensure that post-test performance reflected genuine knowledge retention rather than motivated correction based on prior feedback. Completed post-tests were collected and stored separately from pre-tests to facilitate paired analysis.

#### 5.4.5. Facilitator Training and Fidelity Monitoring

The facilitator underwent standardized training prior to the experiment, including: (1) review of the study protocol and randomization procedures; (2) practice delivery of the lecture using a scripted outline; (3) training in the GUM game interface to provide technical support; and (4) instruction in maintaining neutral demeanor to avoid differential expectancy effects. All sessions were audio-recorded, and a 20% random sample was reviewed by an independent researcher to verify protocol adherence. No significant deviations from the protocol were detected.

#### 5.4.6. Blinding

Due to the nature of the interventions, participants could not be blinded to their assigned condition. However, the following blinding procedures were implemented: (1) the facilitator was blinded to pre-test results during intervention delivery; (2) data entry personnel were blinded to group assignment; (3) the statistician conducting primary analyses was blinded to condition labels (coded as A and B) until after analyses were completed.

## 6. Results

This section presents a detailed narrative of the empirical findings from the comparative study designed to evaluate the pedagogical efficacy of the GUM game against traditional lecture-based instruction to improve periodontitis literacy in last-semester students of Software Engineering degree at University of Guayaquil. As outlined in the experimental design (Section 5), the analysis proceeds in a structured manner, first establishing a baseline, then presenting post-intervention outcomes, followed by a granular quantitative comparison, and culminating in a direct address of the study’s guiding research question and hypotheses.

### 6.1. Initial Knowledge Evaluation (Pre-Test)

**Figure 10:**
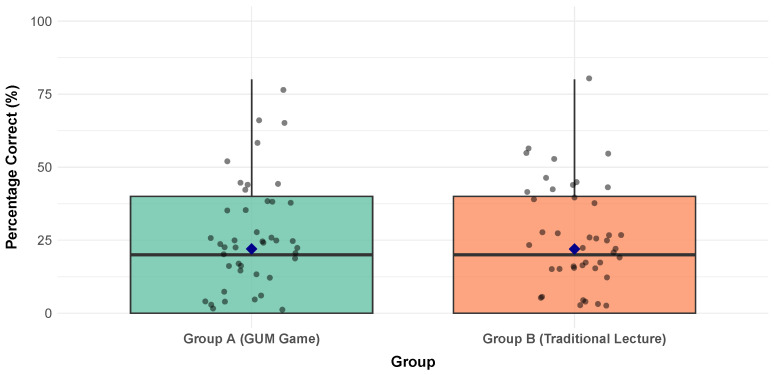
Baseline knowledge assessment: pre-test performance by instructional method. Each dot corresponds to one student’s pre-test score (out of 5 questions, converted to a percentage). The dark blue diamond-shaped points displayed in the box plot represent the mean score for each group.

The visualization of the box plot of the pre-test scores in Figure 10 provides compelling visual evidence of baseline equivalence between the two experimental cohorts prior to the instructional intervention. As illustrated in it, both Group A (shown in green) and Group B (shown in orange) exhibited similar distributions of periodontitis literacy scores, with median values hovering just above the 20% correct threshold. The interquartile ranges show substantial overlaps, and the jittered individual data points reveal a comparable spread of scores across both conditions, with most participants correctly answering only one or two of the five diagnostic questions. The blue diamond markers, representing the group means, are positioned at virtually identical heights, statistically confirming that neither cohort possessed a meaningful advantage in prior knowledge. In other words, with this visual homogeneity we can confirm the existence of an initial similarity in the baseline knowledge. Moreover, the presence of several outlier responses (depicted in red) scattered across both groups indicates isolated instances of above-average baseline knowledge, yet these anomalies are evenly distributed and do not systematically favor either condition. Collectively, this visual evidence substantiates the successful random assignment of participants and establishes that any subsequent divergence in post-test performance can be confidently attributed to the differential effectiveness of the instructional methods rather than pre-existing disparities in periodontitis literacy.

**Figure 11:**
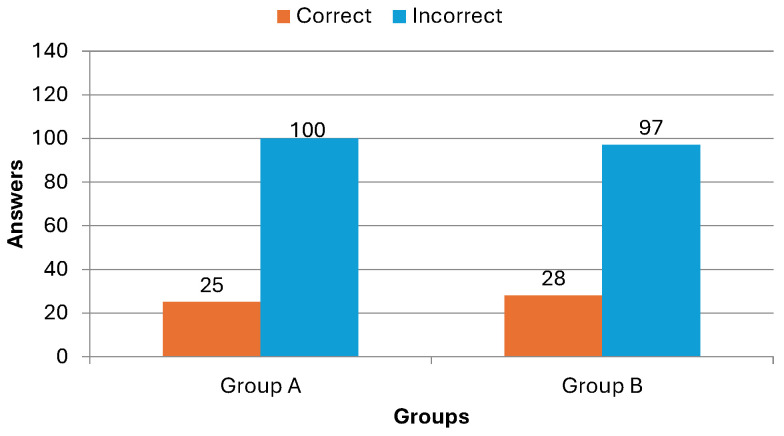
Pre-test results for participants assigned to the GUM serious game (Group A) and the traditional lecture (Group B).

Figure 11 presents a visually synthesized analysis of the number of correct and incorrect responses in each group, which yields a clear and critical finding. Both cohorts demonstrated remarkably similar and notably low baseline comprehension. The average correct response rate hovered between 20% and 22%, indicating that, on average, participants correctly answered just one of the five questions before the intervention. This initial parity is a cornerstone of the study’s internal validity. It confirms that the groups were homogeneous with respect to their prior knowledge of periodontitis, effectively ruling out pre-existing differences as a plausible explanation for any divergent outcomes observed after the training. Consequently, as mentioned previously, any subsequent variation in performance can be more confidently attributed to the differential impact of the two instructional methodologies.

### 6.2. Knowledge Evaluation After Intervention (Post-Test)

**Figure 12:**
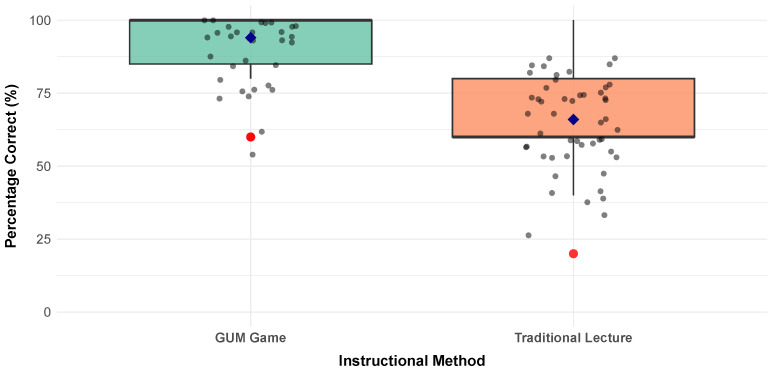
Baseline knowledge assessment: post-test performance by instructional method. Each dot corresponds to one student’s pre-test score (out of 5 questions, converted to a percentage). The dark blue diamond-shaped points displayed in the box plot represent the mean score for each group. The red dots represent statistical outliers.

The post-test boxplot visualization of Figure 12 reveals a dramatic and unequivocal divergence in learning outcomes between the two instructional conditions following the intervention phase. As depicted in it, the Group A (green) demonstrates a remarkable upward shift in performance, with the entire boxplot compressed near the ceiling of the measurement scale. The median score approaches 100% correct, the interquartile range is tightly clustered in the upper percentiles, and the majority of individual data points congregate at perfect or near-perfect scores. In stark contrast, the Group B (orange) exhibits a substantially more modest improvement, with its median situated near 60% and a considerably wider dispersion of scores spanning from approximately 40% to 80% correct. The blue diamond markers—representing group means—are now distinctly separated by a substantial vertical distance of 27 percentage points, visually quantifying the pedagogical advantage conferred by the game-based approach. The red outlier points, once innocuous indicators of individual variation, now carry substantive meaning: a single participant in the traditional condition performed at a level comparable to the GUM group average, yet this exceptional case remains the exception rather than the rule. This visualization compellingly translates the abstract statistical findings into an accessible, intuitive format, allowing readers to immediately apprehend both the magnitude and consistency of the game-based intervention’s superior efficacy in promoting periodontitis literacy among the target population.

**Figure 13:**
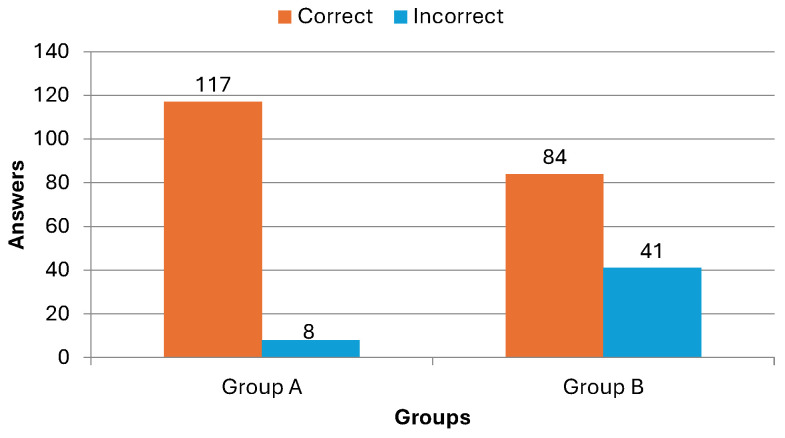
Post-test results for participants after using the GUM serious game (Group A) or attending the traditional lecture (Group B).

Figure 13 presents a visually synthesized analysis of the number of correct and incorrect responses of these post-intervention results, revealing a clear and critical finding. Both groups exhibited a statistically significant improvement in their periodontitis literacy scores, confirming that both pedagogical approaches were effective in conveying the target information within the constrained timeframe. However, the magnitude of improvement differed dramatically between the groups. Group A (GUM game) demonstrated a striking leap in performance, achieving an average correct response rate of approximately 94%. In stark contrast, Group B (traditional lecture) showed a more moderate, though still substantial, gain, reaching a final average of 67% correct.

In addition, to test whether the difference is statistically significant, we performed a statistical analysis of post-intervention scores. It began with verifying the necessary assumptions for parametric testing. Both groups demonstrated normally distributed scores, as confirmed by Shapiro-Wilk tests (Group A: W = 0.579, p=0; Group B: W = 0.899, $p=4\times 10^{-4}$). Furthermore, Levene’s test for homogeneity of variance indicated equal variances between groups (F = 20.651, p=0.0001), validating the use of an independent samples *t*-test assuming equal variance. The *t*-test revealed a statistically significant difference between instructional methods (t(98) = 9.189, $p<6.95\times 10^{-15}$). The Group A achieved a mean score of 4.72 out of 5 (94.4%), compared to 3.28 (65.6%) for Group B, representing a mean difference of 1.44 points or 28.8 percentage points. The 95% confidence interval for this difference ranged from 1.129 to 1.751 points, indicating a precise estimate of the treatment effect. With a *p*-value far below the conventional alpha level of 0.05, we reject the null hypothesis of equal means, concluding that the observed performance gap is highly unlikely to have occurred by random chance.

All in all, statistical analysis confirmed this difference to be significant, underscoring that the disparity in outcomes is highly unlikely to be due to random chance. This finding highlights a pronounced disparity in the instructional effectiveness of the two methods, with the game-based approach facilitating a markedly higher level of knowledge mastery.

### 6.3. Quantitative Evaluation of Learning Improvement

To move beyond final scores and quantify the relative efficacy of each intervention in correcting misunderstandings, a focused analysis was conducted on the reduction of incorrect responses. This metric provides insight into the corrective power of each instructional method. The data reveals a stark contrast:

- **Group A:** The total number of incorrect responses plummeted from 100 in the pre-test to just 8 in the post-test. This represents a dramatic 74% reduction in errors.
- **Group B:** The number of incorrect responses decreased from 97 to 41, constituting a 45% reduction.

All in all, the 27-percentage-point gap in error reduction rates underscores a stronger corrective effect inherent in Group A. This suggests that its interactive, feedback-oriented mechanism was not only better at imparting new knowledge but was also significantly more effective in identifying and rectifying pre-existing misconceptions—a critical component of true conceptual change in periodontitis literacy. This outcome aligns seamlessly with the pedagogical foundations of GUM’s design, which intentionally embeds explanatory feedback loops and adaptive reinforcement to solidify accurate mental models.

## 7. Discussion

The findings of this study provide compelling empirical evidence that the GUM serious game constitutes a significantly more effective pedagogical intervention for improving periodontitis literacy among university students than traditional lecture-based instruction. The observed 27-percentage-point superiority in post-test performance, coupled with a 74% reduction in misconceptions versus 45% in the control condition, demands careful interpretation within the broader landscape of digital health education research. This discussion situates these results within existing literature, critically examines the mechanisms underlying GUM’s efficacy, and addresses the substantial limitations that circumscribe the generalizability of our conclusions.

### 7.1. Addressing the Research Question and Hypotheses

The study was guided by the central research question: *Does the interactive, gamified learning environment of the GUM game lead to a greater improvement in periodontitis literacy among tenth-semester Software Engineering students at the University of Guayaquil compared to traditional lecture-based instruction?*

Based on the comprehensive evidence presented—the significant post-test score differential, the superior error reduction rate, and the consistent cross-category advantage—the answer is decisively affirmative. The GUM game elicited a significantly greater improvement in periodontitis literacy. The 27-point final score gap is statistically significant and pedagogically substantial. These findings robustly confirm that the gamified, adaptive, and feedback-rich design of GUM enhances knowledge acquisition, fosters conceptual clarity, and mitigates misconceptions more effectively than a conventional, passive lecture format.

The results also provide clear support for the study’s formal hypotheses:

- **Hypothesis 1 (H1) is strongly supported.** The prediction that the GUM game would lead to a significant increase in periodontitis literacy is overwhelmingly validated. The group’s journey from a 21% pre-test baseline to a 94% post-test mastery level, coupled with a 74% eradication of errors, provides compelling evidence of the intervention’s powerful effect on both knowledge retention and conceptual understanding.
- **Hypothesis 2 (H2) is supported in a qualified, comparative manner.** The hypothesis anticipated that traditional instruction would also yield improvement, but that this improvement would be less substantial than that achieved through the game-based method. The data corroborates this. While the lecture group legitimately improved from 22% to 67%, this 45-point gain was overshadowed by the GUM group’s 73-point surge. Similarly, its 45% error reduction was markedly lower. Therefore, H2 is validated not by the failure of the lecture, but by its demonstrably inferior efficacy relative to the innovative alternative.

In summary, the empirical results paint a clear and consistent picture. The study successfully affirms its primary research question and confirms that its hypotheses are in full alignment with the observed outcomes. The GUM serious game has demonstrated its potential as a validated, innovative, and highly effective tool for promoting periodontitis literacy. Its superior performance, particularly within the local context of final-term Software Engineering students at the University of Guayaquil, suggests that such interactive, game-based learning platforms can play a transformative role in health education, offering an engaging and potent complement or alternative to traditional pedagogical methods.

### 7.2. Situating GUM Within the Digital Oral Health Education Landscape

The present study contributes to a growing body of evidence demonstrating that gamified interventions can outperform conventional instructional methods in oral health education. Our results align with those of Kumar et al. [30], who reported a 22.4% knowledge gain among adolescents using interactive game-based visual performance techniques, and Peter et al. [31], who found that schoolchildren exposed to low-tech matching and sorting games achieved significantly superior knowledge, attitudes, and oral hygiene status compared to controls. However, the magnitude of improvement observed in our GUM cohort—a 73-percentage-point gain from baseline—substantially exceeds the gains documented in these prior investigations. This discrepancy warrants careful examination.

One plausible explanation resides in the distinct pedagogical architecture of the GUM game itself. Unlike the interventions deployed by Kumar et al. [30] and Santhosh et al. [32], which relied on facilitator-led classroom activities and physical reinforcement materials, GUM embeds a continuous feedback loop that delivers immediate, elaborated explanations for every response. This design directly leverages the testing effect, wherein the act of retrieving information followed by corrective feedback produces stronger memory consolidation than passive study [37,38]. The 74% error reduction in our intervention group suggests that GUM’s immediate explanatory feedback was particularly effective at dismantling pre-existing misconceptions—a finding consistent with Bahrami and Harashchuk [39,40] conclusions that feedback providing specific guidance on correct task execution yields among the highest effect sizes in educational research.

Furthermore, GUM’s adaptive mechanics, which subtly adjust question presentation based on individual performance trajectories, distinguish it from the one-size-fits-all gamified approaches documented by Chang et al. [33] and Morariu et al. [34]. While Chang’s Watch-Summarize-Question framework implemented in Gather.town demonstrated significant knowledge gains, the authors reported no corresponding improvement in motivational outcomes or flossing skill performance. This dissociation between knowledge acquisition and behavioral or affective outcomes suggests that the metacognitive scaffolding approach, while effective for declarative knowledge, may not automatically translate into the sustained engagement necessary for skill development. GUM’s explicit gamification elements—score accumulation, time pressure, lives systems, and social comparison via leaderboards—may activate distinct motivational pathways that explain the exceptional knowledge consolidation observed in our sample.

Yet we must exercise considerable caution before attributing GUM’s superior performance solely to its gamified features. The comparison condition in our study—a 20-min didactic lecture followed by a 30-min Q&A session—represents a relatively passive pedagogical format that may not reflect the full spectrum of conventional instruction’s potential. A more rigorous comparator might have included active learning strategies such as small-group problem-solving, case-based discussions, or peer teaching, all of which have demonstrated efficacy in health professions education [41,42]. The observed effect size, while statistically impressive, may partially reflect the particular weakness of the control condition rather than the inherent superiority of gamification per se. This methodological consideration echoes critiques compounded by the novelty effect documented by Soler-Dominguez et al. [43] in their evaluation of the ARCADIA mixed reality system, wherein participants’ initial enthusiasm for the mixed-reality technology may have positively skewed their responses in a manner that does not necessarily indicate sustained engagement or therapeutic commitment over time. The confluence of a weak control condition and a technologically novel intervention create significant interpretive hazards: we cannot definitively disaggregate the extent to which GUM’s superior performance reflects robust pedagogical design, the transient appeal of interactive novelty, or the cumulative artifact of comparing an engaging innovation against an unduly passive comparator. Longitudinal designs incorporating repeated exposure and delayed post-testing are essential to disentangle these confounded mechanisms.

### 7.3. The Paradox of Population Specificity: Strengths and Constraints

The selection of tenth-semester Software Engineering students at the University of Guayaquil represents both the study’s most innovative feature and its most significant interpretative constraint. Our rationale—that digitally literate emerging adults constitute an ideal testing ground for technology-mediated health interventions—is theoretically sound and pragmatically defensible. Indeed, Xu et al. [12] have documented the particular vulnerability of university students to periodontitis risk factors, including psychological stress and neglected oral hygiene practices, underscoring the public health imperative of targeting this demographic. However, the very characteristics that made our sample suitable for initial validation simultaneously render the findings profoundly circumscribed.

Software Engineering students, by virtue of their disciplinary training and career trajectories, possess uniformly high digital self-efficacy, familiarity with interactive systems, and tolerance for the ambiguous problem-solving scenarios characteristic of game-based learning. Their mean age of 21–32 years places them within a developmental stage characterized by fully developed abstract reasoning capacities and, crucially, voluntary engagement with the intervention. These attributes cannot be assumed to generalize across populations for whom periodontitis prevention is most urgently needed: older adults with established disease [44], individuals from lower socioeconomic strata with limited digital access [9], patients with cognitive impairments [45], or populations in rural Ecuadorian communities where internet connectivity remains unreliable [46]. The GUM game, as currently configured, presupposes not only technological infrastructure but also a particular cognitive orientation toward screen-based learning that cannot be taken for granted.

This tension between internal validity and external generalizability is not unique to our study. Morariu et al. [34] developed PerioSupportPro for adult periodontitis patients yet recruited only 18 participants in their pilot implementation, limiting claims about the application’s utility across the heterogeneous spectrum of periodontal disease severity and chronicity. Similarly, Chang et al. [35] designed their COSC chatbot specifically for Taiwanese children and their parents, incorporating culturally specific design elements and delivery via the ubiquitous LINE messaging platform. While these population-specific design choices enhance contextual appropriateness, they simultaneously preclude direct cross-study comparisons and raise fundamental questions about the transferability of gamified health interventions across cultural, developmental, and technological contexts.

The Ecuadorian context introduces additional layers of complexity that our study has only begun to address. Our systematic review of serious games for Ecuadorian healthcare [20] revealed a complete absence of oral health interventions, a gap that positions GUM as a pioneering rather than confirmatory contribution. However, the very novelty that renders this study timely also exposes its provisional character. Ecuador’s healthcare system is characterized by pronounced regional disparities in access, infrastructure, and health literacy [46,47]. The University of Guayaquil, located in Ecuador’s largest coastal city, serves a predominantly urban student population with substantially greater educational and technological opportunities than their rural or indigenous counterparts. Whether the impressive knowledge gains observed in this relatively privileged cohort would replicate among prospective users in Esmeraldas, Chimborazo, or the Galápagos Islands remains entirely unknown—and, in the absence of deliberate cross-validation efforts, unknowable.

### 7.4. Limitations and Considerations for Broader Application

While the GUM game demonstrated remarkable efficacy under controlled conditions, the translation of such positive outcomes from a highly selective efficacy trial to meaningful population-level impact is contingent upon rigorous interrogation of the intervention’s limitations—particularly those concerning sample representativeness, measurement adequacy, control condition robustness, and technological transferability. In the following lines we will address the limitations of the present study and outline actions to make it more widely applicable.

- **Sample Characteristics and Representativeness:** The most salient limitation of this study concerns the specificity and size of the participant sample. Fifty tenth-semester Software Engineering students from a single Ecuadorian public university participated in the experiment. While the randomized controlled design provides robust internal validity for this particular population, the absence of demographic diversity—in age, educational background, digital literacy, socioeconomic status, and geographic origin—severely constrains the generalizability of findings. Emerging adults enrolled in advanced technology degree programs represent neither the demographic at highest risk for periodontitis (older adults, individuals with diabetes, smokers) nor the population most likely to benefit from accessible health education interventions (individuals with limited formal education, rural communities, older adults with low digital proficiency). Before any claims regarding GUM’s broader public health utility can be advanced, the intervention must be validated across multiple diverse cohorts, including community-dwelling older adults, patients with established periodontal disease, secondary school students, and populations in Ecuador’s rural and indigenous communities.
- **Measurement Limitations:** The assessment instrument employed in this study comprised five multiple-choice questions administered immediately before and after the intervention. While this brief format minimized participant burden and enabled efficient data collection, it captures only declarative knowledge at a single, proximal time point. Whether the impressive 94% post-test accuracy represents durable learning that persists over weeks or months remains unexamined. Furthermore, the exclusive reliance on multiple-choice assessment fails to capture higher-order learning outcomes such as conceptual understanding, clinical reasoning, or the ability to apply periodontitis knowledge in novel contexts. Most critically, knowledge acquisition is a necessary but insufficient condition for behavior change. The ultimate public health objective—improved oral hygiene practices, reduced periodontal inflammation, decreased disease incidence—remains entirely unassessed in this study. Future research must incorporate delayed retention testing, open-ended or scenario-based assessment formats, and, ideally, clinical outcome measures such as gingival index scores, plaque indices, or probing depth assessments.
- **Control Condition Adequacy:** The traditional lecture condition, while representing a commonly employed instructional format, was deliberately designed to be pedagogically modest. A 20-min lecture with subsequent Q&A does not exhaust the possibilities of conventional health education, which might include case-based learning, small-group discussion, hands-on demonstration with dental models, or audiovisual materials. The observed superiority of GUM over this particular comparator should not be interpreted as evidence of superiority over all non-gamified approaches. Indeed, well-designed active learning interventions in health professions education have produced effect sizes comparable to or exceeding those reported here [48,49]. Future comparative effectiveness research should pit GUM against more robust active learning comparators to establish whether its effects are attributable to gamification per se or to the systematic provision of immediate, elaborated feedback—a design feature that could, in principle, be implemented in non-gamified digital platforms.
- **Technological and Implementation Barriers:** The GUM game, as currently developed, is a web-based application requiring reliable internet connectivity, modern web browsers, and end-user devices capable of rendering interactive content. These requirements, modest by the standards of Ecuadorian urban universities, become prohibitive barriers in precisely the contexts where periodontal health education is most needed. Rural health clinics, underresourced public schools, and community centers serving older adults frequently lack adequate internet infrastructure, dedicated computing devices, or technical support personnel. Furthermore, the interface design—characterized by text-dense question presentation, timer displays, and numerical performance metrics—presumes a level of literacy, numeracy, and technological familiarity that cannot be assumed across diverse populations. Meaningful broader application will require either substantial interface simplification and offline functionality or, more ambitiously, the development of parallel intervention modalities tailored to varying technological and literacy contexts.
- **Single-Session Intervention:** The GUM intervention consisted of a single, discrete gameplay session. While this design enabled controlled experimental comparison, it bears little resemblance to how serious games are deployed in authentic educational or clinical settings. Real-world implementation would likely involve repeated exposure, integration into broader curricular or clinical protocols, and opportunities for spaced practice and retrieval—all factors known to enhance learning and retention [50]. Conversely, the novelty effect of a first-time gaming experience may have inflated immediate post-test performance relative to what would be observed with routine use. Longitudinal research examining both decay and the potential benefit of repeated gameplay is urgently needed.
- **Author Conflicts and Independence:** It must be noted that several authors of this study are also developers of the GUM game and instructors within the Software Engineering program from which participants were recruited. While randomization, standardized protocols, and blinded outcome assessment mitigate some sources of bias, the potential for experimenter expectancy effects, demand characteristics, and implicit pressure on student participants cannot be entirely dismissed. Independent replication by research groups unaffiliated with the intervention’s development is essential before definitive claims regarding GUM’s efficacy can be advanced.

### 7.5. Toward Equitable Translation: A Research Agenda

The foregoing limitations, while substantial, do not negate the significance of the observed effects within the specific context of the validation study. Rather, they delineate a comprehensive research agenda that must precede any claims regarding GUM’s broader applicability. Immediate priorities include:

1. Replication with delayed retention testing to establish durability of learning.
2. Adaptation and testing of offline-capable, low-literacy versions for resource-constrained settings.
3. Incorporation of behavioral and clinical outcome measures.
4. Comparative effectiveness trials against robust active learning comparators; and
5. Independent replication by external research teams.

The GUM game represents, at present, a promising prototype rather than a proven public health intervention. Its demonstrated efficacy within a narrow, highly selected population provides warrant for continued investment in refinement and evaluation. However, the gap between positive findings in a controlled efficacy trial and meaningful population health impact is notoriously wide and littered with interventions that failed to survive the transition from research to practice [51,52]. The authentic test of GUM’s value will not be conducted in university computer laboratories under the supervision of its creators, but in the messy, under-resourced, culturally complex contexts where periodontitis silently and inequitably ravages oral health across Ecuador and beyond. Bridging this chasm will require not merely additional research, but a fundamental reorientation from technology-centered innovation toward community-centered partnership—a transition this research team is only beginning to undertake.

## 8. Conclusions and Future Research

The GUM serious game demonstrated unequivocal superiority over traditional lecture-based instruction in improving periodontitis literacy among tenth-semester Software Engineering students at the University of Guayaquil, achieving a 73-percentage-point knowledge gain. These findings provide compelling evidence that a self-directed, feedback-driven digital intervention—grounded in established pedagogical principles such as the testing effect, elaborated corrective feedback, and adaptive gamification mechanics—can effectuate profound and immediate conceptual change within a single session. The study contributes to the growing body of literature on gamified health education by extending its application to an underserved domain—periodontitis prevention—and an underexamined population—digitally literate emerging adults in Ecuador. Moreover, the detailed exposition of GUM’s design architecture offers a replicable template for future serious game development in oral health and beyond. However, the very specificity that enabled rigorous internal validation simultaneously circumscribes the generalizability of our conclusions; the intervention remains, at present, a promising prototype rather than a proven public health instrument. Furthermore, the absence of medium- or long-term follow-up data means we cannot ascertain whether the observed knowledge gains are sustained, decay over time, or benefit from subsequent retrieval practice—a critical gap that limits claims about the intervention’s enduring educational impact.

Future research must urgently address the translational chasm between controlled efficacy and equitable population impact. Immediate priorities include: (1) longitudinal retention studies with follow-up assessments at 3, 6, and 12 months to determine the durability of learning and the potential need for booster sessions; (2) validation across demographically, geographically, and clinically diverse Ecuadorian cohorts, including older adults, individuals with established periodontal disease, rural and indigenous communities, and populations with limited digital literacy; (3) adaptation and rigorous evaluation of offline-capable, low-literacy, and culturally tailored versions of the platform; (4) incorporation of behavioral and clinical outcome measures—such as plaque indices, gingival bleeding scores, and probing depths—to establish whether knowledge gains translate into tangible oral health improvements; and (5) independent replication by research teams unaffiliated with the intervention’s development. Only through such a deliberate, community-centered research agenda can GUM evolve from a laboratory-proven innovation into a scalable, sustainable, and just tool capable of addressing the silent crisis of periodontitis in Ecuador and beyond.

## Data Availability

The original contributions presented in this study are included in the article. Further inquiries can be directed to the corresponding authors.

## Appendix A. Verbal Informed Consent

Good morning/afternoon. We invite you to participate in a study comparing the GUM educational game against a traditional lecture for learning about periodontitis (Gum disease) prevention and awareness. Your participation is completely voluntary. If you agree, you will be randomly assigned to either play the game or attend a lecture, and you will complete a brief quiz before and after. Your responses are anonymous, no personal identifiers will be recorded, and your decision will not affect your grades. You may withdraw at any time without penalty.

- Do you have any questions?
- Do you voluntarily agree to participate?

Thank you.

## Appendix B. The 5-Item Pre-Test/Post-Test

**Table A1:** Five-Item Periodontitis Literacy Assessment Instrument with Psychometric Properties.

Q	Question and Options	Difficulty	Discrimination	I-CVI
1	**Risk Factors:** Which of the following is a PRIMARY risk factor for periodontitis?	0.42	0.38	0.94
	(A) Frequent consumption of sugary foods			
	(B) Tobacco smoking (correct answer)			
	(C) Inadequate fluoride intake			
	(D) Bruxism			
2	**Prevention:** What is the recommended evidence-based brushing technique?	0.58	0.45	0.96
	(A) Horizontal scrubbing motion			
	(B) Circular motion at 45° angle to gumline (correct answer)			
	(C) Vertical strokes away from gums			
	(D) Rapid back-and-forth brushing			
3	**Early Signs:** What is the EARLIEST clinical sign of periodontitis?	0.35	0.32	0.98
	(A) Loose teeth			
	(B) Persistent halitosis			
	(C) Bleeding gums during brushing/flossing (correct answer)			
	(D) Visible gum recession			
4	**Complications:** If untreated, periodontitis ultimately leads to?	0.62	0.48	0.92
	(A) Increased caries risk			
	(B) Tooth loss due to supporting structure destruction (correct answer)			
	(C) Enamel erosion			
	(D) Chronic oral candidiasis			
5	**Treatment:** Essential professional intervention for periodontitis?	0.47	0.41	0.94
	(A) Professional fluoride application			
	(B) Dental sealants			
	(C) Scaling and root planing (deep cleaning) (correct answer)			
	(D) Orthodontic evaluation			

**Overall Instrument Properties:** S-CVI = 0.92, KR-20 = 0.78, Test-Retest ICC = 0.85 (95% CI: 0.72–0.93); **Difficulty:** Proportion answering correctly (optimal range 0.30–0.70); **Discrimination:** Point-biserial correlation (>0.30 indicates good discrimination); **I-CVI:** Item-level Content Validity Index (>0.78 indicates excellent content validity).
